# A Study of the Reliability and Validity of the Korean Version of the Penn Alcohol Craving Scale for Alcohol-Dependent Patients

**DOI:** 10.4306/pi.2008.5.3.175

**Published:** 2008-09-30

**Authors:** Min Jeong Kim, Sung Gon Kim, Hyo Jeong Kim, Ho Chan Kim, Ji Heh Park, Kwang Seok Park, Duk Ki Lee, Won Tan Byun, Cheol Min Kim

**Affiliations:** 1 Department of Psychiatry, School of Medicine, Pusan National University, Busan, Korea.; 2 Department of Psychiatry, Yang San Hospital, Yangsan, Korea.; 3 Department of Psychiatry, College of Medicine, Kosin University, Busan, Korea.; 4 School of Medicine, Pusan National University, Busan, Korea.; 5 Department of Psychiatry, Yang San Hae-In General Hospital, Yangsan, Korea.

**Keywords:** Korean version of the Penn Alcohol Craving Scale, Reliability, Validity, Alcohol

## Abstract

**Objective:**

The Penn Alcohol Craving Scale (PACS) is a stronger predictor of subsequent drinking and relapse of alcohol dependence that can be administered more quickly and easily than other craving scales. The goal of this study was to develop the Korean version of the Penn Alcohol Craving Scale (PACS-K).

**Methods:**

To examine the psychometric properties of the PACS-K, responses were chosen from 80 patients admitted to a treatment facility for alcohol dependence.

**Results:**

The PACS-K possesses good psychometric properties, as assessed by Cronbach's α estimates (Cronbach's α=0.91). The test-retest reliability of the PACS-K showed high correlation (p<0.01) when the retest interval was 1 day. When the validity of the PACS-K was investigated using correlation analysis with two other craving scales (the Obsessive Compulsive Drinking Scale (OCDS) and the Visual Analogue Scale (VAS), high correlations were obtained between total PACS scores and total OCDS scores, and between total PACS scores and VAS scores (p<0.01, respectively).

**Conclusion:**

The PACS-K is a reliable and valid measure of alcohol cravings, and it could be useful for predicting which individuals are at risk for subsequent relapse.

## Introduction

Alcohol dependent disorder has one of the highest rates of relapse among psychiatric disorders. Therefore, the prediction and prevention of relapse of alcohol dependence represent a very important part of treatment. In treating alcohol dependence, the degree of alcohol craving is known to affect the maintenance of abstinence and drinking again after a period of abstinence.^1^-^3^ Furthermore, studies assessing the effects of long term treatment of alcohol dependence showed that alcohol craving was related to relapse, and that craving represented a response to the loss of control of drinking, internal temptation, and exposure to stimuli, etc.^4^ Therefore, alcohol craving is the most important factor of relapse of alcohol dependence, and measuring the degree of alcohol craving is important in preventing relapse of alcohol dependence.^5^

The Visual Analogue Scale (VAS) has been used to measure the degree of alcohol craving, and tools like the Obsessive-Compulsive Drinking Scale (OCDS),^6^ Alcohol Urge Questionnaire (AUQ),^7^ etc. have already been developed and are currently being used. However, they contain items that measure aspects other than alcohol craving^7^ or have limitations in that they contain too many questions and take too long to be applied during the limited time available in the outpatient treatment setting. In addition, a single-item scale like the VAS, which is a simple tool that measures alcohol craving, can be administered easily and shows good consistency. However, with the VAS did not have good predictive validity, and confusion as to the meaning of craving can arise, as it is not clearly defined in the VAS.^4^,^5^,^7^ The 5-item Penn Alcohol Craving Scale (PACS) was developed by improving these shortcomings.^5^ The PACS provides a more direct expression of craving from the subject's point of view, and it is a multiple item, single factor scale used to measure alcohol craving that can be assessed in a short period of time.

The PACS consists of 3 items that assess the frequency, intensity and duration of alcohol cravings, an item measuring the subject's ability to resist temptation when drinking is possible, and an item assessing the degree of general alcohol craving over the past week. The respondents can rate each of the 5 items on a scale from 0 to 6.^5^ The degree to which alcohol cravings during the treatment period of alcohol dependence is related to the subsequent relapse was investigated in order to determine whether the PACS can predict the degree of alcohol craving that influences the relapse of alcohol dependence. The results revealed that the degree of alcohol craving measured by the PACS predicts relapse after 1 week of treatment with validity, and its predictive validity was higher than those of the Obsessive subscale (OBS) of the OCDS and the AUQ.^8^

Therefore, this study intends to develop the Korean Penn Alcohol Craving Scale (PACS-K) to efficiently measure alcohol cravings in clinical settings by translating the PACS with these merits into Korean so that it can be first used in Korea and then verify its reliability and validity. The PACS-K can be easily used to measure alcohol craving in treatment and studies of alcohol dependence in Korea, and it will be able to provide a good predictive factor for relapse of alcohol dependence.

## Methods

### Study subjects

This study targeted patients with alcohol dependence aged over 20 and less than 65 years who were hospitalized in psychiatric wards from April 2007 to September 2007. The diagnosis of alcohol dependence was made by 2 psychiatrists based on the Diagnostic and Statistical Manual of Mental Disorders, Fourth Edition (DSM-IV).^9^ Patients with strong denial of alcohol dependence or severe cognitive disorders, patients with severe withdrawal symptoms and patients with other Axis I disorders were excluded. In addition, after examining the questionnaires completed by the subjects, subjects for whom the credibility of the data was dubious were excluded. The number of subjects included in the final study was 80.

Subjects' basic data, such as socio-demographic characteristics and alcohol history, were assessed based on structured interviews with the subjects.

### Procedure

After obtaining basic data by interviewing the subjects who provided informed consent to the procedures of this study, test sheets were distributed in the following order: VAS1, PACS-K, VAS2 and OCDS, and they were read and answered by the subjects themselves. After examining whether the PACS-K is reliable as a measuring tool by determining the internal consistency of the PACS-K, test-retest reliability verification was performed to secure higher reliability. To verify the test-retest reliability of the VAS and the PACS-K, the tests were administered twice (with a 1-day interval) to patients hospitalized in psychiatric wards, excluding the subjects who were previously participated on verifying the validity of PACS-K. Test-retest reliability was verified in the final 20 patients, except for non-respondents and those with dubious reliability.

#### Korean Penn Alcohol Craving Scale

The Korean translated version of the original English version of the PACS (the PACS-K) was used in this study. A psychiatrist who was familiar with the English terms related to alcohol dependence treatment translated the tool, and items that might fail to deliver an accurate meaning when directly translated due to cultural differnces were culturally adapted. After the draft was finalized, a psychiatrist, psychiatric resident, nurse and psychologist discussed the content of the translated version, modified it, tried it in several cases and then re-modified it. The final version of the PACS-K has a total of 5 items, and each item is structured to measure the degree of alcohol craving on a scale from 0 to 6.

#### Obsessive Compulsive Drinking Scale

The OCDS is a self-report questionnaire consisting of 14 items,^6^ and the Korean version^10^ was used in this study. The OCDS measures obsessive thinking about alcohol and the obsessive use of alcohol. The internal consistency (Cronbach's α) of the Korean version of the OCDS is 0.89.^10^

#### Visual Analogue Scale

The VAS uses a 100-mm line with items of 'Do not want to drink at all' and 'Want to drink very much' at the two axes in order to measure how much the subject presently wants to drink. The VAS was appraised twice in order to prevent the bias in the responses of the subject as well as random responses. Data with significant differences between the VAS1 and VAS2 responses were excluded from the data analysis.

### Data analysis

All statistical analyses were performed using SPSS PC+ version 11.5. The internal consistency used to verify the reliability of the PACS-K was measured by determining the Cronbach's α coefficients, and verification of test-retest reliability and validity was measured by Pearson correlation coefficients.

## Results

### Socio-demographic properties

Table 1 presents the socio-demographic characteristics of the 80 subjects who were included in the final study analysis.

The subjects were all male, with an average age of 46.6±7.4 years. The mean years of education was 10.5±3.4, 31.6% of the subjects were married, and 67.1% of the subjects had an occupation. The average age at which the subjects started drinking was 19.0±4.0 years, the average age at onset of alcohol-related problems was 32.5±9.3 years, and the average frequency of hospitalization was 8.3±13.0 times. The average number of drinking days per month within the past year was 18.0±10.9 days, and the average amount of alcohol consumed per drinking day in the past year was 12.5±6.3 standard drinks (SD). A family history of alcohol dependence was noted in 33.8% of the subjects, and 68.8% had experienced withdrawal symptoms (Table 1).

### Reliability

The results of internal consistency analysis of the 5 items of the PACS-K showed that the Cronbach's α coefficient was 0.91. The PACS-K showed high internal consistency, which confirmed its reliability.

The results of the PACS-K, performed at 1-day intervals to verify its test-retest reliability, showed that there is a significantly high degree of correlation between the baseline and PACS-K result after 1 day (r=0.769, p<0.01).

### Validity

The results of correlation analysis of the VAS and OCDS showed that both scales are significantly associated with the PACS-K (r=0.664 with VAS1, r=0.569 with VAS2, r=0.509 with OCDS, p<0.01)(Table 2).

## Discussion

To summarize the results of this study, the PACS-K has good internal consistency (Cronbach's α=0.91), and its internal consistency is similar to that of the PACS (Cronbach's α=0.92), as reported by Flannery et al.^5^ In addition, there was a high correlation in the test-retest reliability measurement of the PACS-K practiced with the 1-day interval, and this implies that the PACS-K is a scale that can be used to measure alcohol craving with good reliability. In addition, the results of a correlation analysis revealed that the PACS-K has a significant correlation with the VAS and the OCDS, enough to be relied on, and this further implies that the PACS-K has sufficient conformity and validity.

The PACS-K used in this study consists of 5 items used to measure alcohol craving, just as the original English scale, the PACS, has the same content construction and measures a single-factor related to alcohol craving. It has 3 items for assessing the frequency, intensity and duration of alcohol cravings. It also includes one item for measuring the subject's ability to resist temptation when drinking is possible, and one item to assess the degree of general alcohol craving over the past week. In addition, just as in the PACS, each of the 5 items of the PACS-K is to be evaluated on a scale from 0 to 6.^5^

When considering the fact that drugs like naltrexone decrease the alcohol cravings of patients undergoing treatment for alcohol dependence,^11^-^13^ measuring the degree of alcohol craving can aid in evaluating the therapeutic results of treatment for alcohol dependence and predict relapse of alcohol dependence. In the studies investigating whether the degree of alcohol craving measured by the English scale, the PACS, predicts subsequent drinking, it was reported that PACS score measured during treatment for alcohol dependence was a better predictor of relapse after 1 week than the obsessive scale of the OCDS or the AUQ, demonstrating the clinical excellence of the PACS with regard to alcohol craving.^8^ Furthermore, limitations of many tools previously developed to measure the degree of alcohol craving, such as causing confusion with regard to the meaning of alcohol craving, measuring aspects other than alcohol craving due to inclusion of items that measure aspects other than alcohol craving^7^, requiring too much time for test completion because they include too many items, etc. can be overcome by using the PACS.

The PACS-K developed in this study has also been demonstrated to be valid and reliable as a clinical tool for measuring alcohol craving. In addition, the PACS-K has the same number of items and structure as the PACS. In addition, like the PACS, it overcomes the shortcomings of previous scales used to measure alcohol dependence, such as the long duration of time required for test completion, many items, measuring items other than alcohol craving, low predictive validity, etc. Especially, in terms of predicting relapse of alcohol dependence, the predictive validity of the PACS during the treatment period was high, and the same effect can be expected from the use of the PACS-K. Ultimately, it is suggested that the PACS-K will be able to measure the alcohol cravings of alcohol-dependent patients treated as inpatients or outpatients efficiently in a short period of time, and further, it is supposed that the PACS-K will help predict relapse in patients with alcohol dependence.

There are two important limitations in this study. First, the 1-day interval of test-retest reliability might have been too short, even though it was an attempt to eliminate the influence of changes in the subjects' characteristics, which may be attributable to the inpatient treatment or the possibility that the patients were hiding their alcohol cravings because they were in the hospital. Re-evaluation of the test-retest reliability with a longer interval would be needed in a future study. Second, the PACS-K is a multi-item, single-factor scale used to measure alcohol cravings; however, the OCDS encompasses dimensions of alcohol dependence other than craving.^5^ In the future, it would be useful to investigate the correlation between the PACS-K and some items of the OCDS used to measure alcohol cravings or another single-factor scale for craving.

## Figures and Tables

**TABLE 1:**
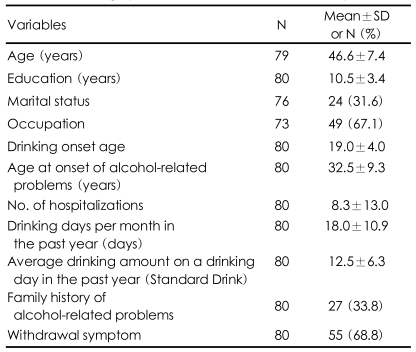
Demographic characteristics of 80 alcoholic patients

**TABLE 2:**
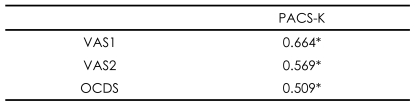
Correlation among the Korean Penn Alcohol Craving Scale (PACS-K), the Visual Analogue Scale (VAS) and the Obsessive Compulsive Drinking Scale (OCDS) (N=80, correlation coefficient)

^*^p<0.01
